# Examining the Efficacy of Antimicrobial Therapy in Preventing the Development of Postinfectious Glomerulonephritis: A Systematic Review and Meta-Analysis

**DOI:** 10.3390/idr14020022

**Published:** 2022-03-07

**Authors:** Emily Bateman, Sara Mansour, Euchariachristy Okafor, Kedzie Arrington, Bo-Young Hong, Jorge Cervantes

**Affiliations:** 1 Paul L Foster School of Medicine, Texas Tech University Health Sciences Center El Paso, El Paso, TX 79905, USA; emily.bateman@ttuhsc.edu (E.B.); or man08916@ttuhsc.edu (S.M.); euokafor@ttuhsc.edu (E.O.); kedzie.arrington@ttuhsc.edu (K.A.); 2 Woody L Hunt School of Dental Medicine, Texas Tech University Health Sciences Center El Paso, El Paso, TX 79905, USA; bo-young.hong@ttuhsc.edu

**Keywords:** postinfectious glomerulonephritis, *Streptococcus pyogenes*, antimicrobial, antibiotic

## Abstract

Postinfectious glomerulonephritis (PIGN) is an immune-mediated acute glomerulonephritis classically seen weeks after infection with *Streptococcus pyogenes*, although other infectious etiologies have emerged. While it has become increasingly rare in industrialized regions, it continues to affect children in developing countries. There has been debate as to why incidence rates are declining, including the possibility of improved initial treatment of bacterial infections. The ability of antimicrobial therapy in preventing PIGN as infectious sequelae, however, has not been comprehensively assessed. As varying evidence from published studies exists, the objective of this meta-analysis is to determine if antimicrobial therapy utilized to treat an initial infection has an effect in reducing the development of PIGN in humans. EMBASE, MEDLINE, and CENTRAL were searched using a comprehensive terminology strategy. From an initial search that returned 337 publications, 9 articles were included for analysis. Eight studies showed an incidence of PIGN after antimicrobial use ranging from 0.05% to 10% with a mean standardized difference (MSD) of 0.03 (0.01–0.06). Three studies showed an occurrence of PIGN without antibiotic use ranging from 1% to 13% with an MSD of 0.06 (−0.09–0.21). Our findings suggest that antimicrobial treatment for the initial infection may help diminish the development of PIGN. Although *Streptococcus pyogenes* infections are generally treated aggressively to prevent rheumatic fever, these findings may help further support the early treatment of bacterial infections to prevent postinfectious sequelae, especially as we consider other infectious etiologies of PIGN antimicrobial resistance.

## 1. Introduction

Postinfectious glomerulonephritis (PIGN) is an acute inflammatory disorder classified as a nephritic syndrome, resulting in hematuria, oliguria, hypertension, and edema when symptomatic [1]. This condition most frequently affects children and generally has a good prognosis [2]. It is classically seen one to two weeks following infection with *Streptococcus pyogenes*, although other infectious etiologies have been identified [3]. For this reason, it has historically been referred to as poststreptococcal glomerulonephritis (PSGN). While the exact mechanism is unknown, it is currently considered a type III hypersensitivity reaction that involves the accumulation of immune antigen–antibody complexes in the glomerulus, triggering an immune response and resulting in tissue damage in the kidney [4,5]. Newer theories also suggest an antibody and T-cell-mediated response to self-antigens due to molecular mimicry [6,7,8].

The majority of recent PIGN cases have affected children and occurred in low socioeconomic countries. In industrialized countries, the incidence of PIGN has become increasingly rare, with most cases occurring from nonstreptococcal bacterial infections in older individuals [9]. The mechanism behind the declining incidence rate is unknown, but it has been proposed that overall improvements in socioeconomic conditions, improved health care access, and altered susceptibility to GAS antigens plays a role. It has also been proposed that improved access to early initial treatment of bacterial infections may also contribute to this decline [10,11,12]. However, this suggestion contrasts the widely taught belief that initial treatment of bacterial infections with antimicrobials does not decrease the risk of developing PIGN [13,14]. The ability of antimicrobial therapies in preventing PIGN as infectious sequelae has not yet been comprehensively assessed, with contradictory evidence from published studies [15].

Establishing the effect of antimicrobials in preventing PIGN is important, especially as we consider new *Streptococcal* spp. strains with reduced susceptibility to broad-spectrum antimicrobials [16,17,18]. The objective of this meta-analysis is to determine the association between antimicrobial therapy use during an initial infection and the subsequent development of PIGN in humans.

## 2. Materials and Methods

### 2.1. Literature Search Strategy

Electronic searches of MEDLINE (all available years to May 2021), EMBASE (all available years to May 2021), and the Cochrane CENTRAL Register (all available years to May 2021) were performed. The terms used in this search were “psgn” OR “pign” OR “postinfectious glomerulonephritis” OR “post-infectious glomerulonephritis” OR “poststrep glomerulonephritis” OR “poststreptococcal glomerulonephritis” OR “post-strep glomerulonephritis” OR “post-streptococcal glomerulonephritis” AND “antibiotic” OR “antimicrobial”. Results were restricted to human studies, and no language restrictions were included. After the initial search, the study was registered in PROSPERO (Registration # CRD42021243989).

### 2.2. Data Collection

Quality of studies was assessed using tools outlined by the National Institutes of Health [19]. These guidelines allowed consideration of different study designs and assessment of risk of bias and are a preferred tool in systematic reviews [20]. After electronic de-duplication, studies were screened by four researchers (E.B., S.M., E.O., and K.A.), who agreed on included studies and achieved consensus on data collected. This allowed for identification of data which may have been omitted by a single reviewer. Screening also allowed for deduplication that did not initially occur electronically. Case studies, reviews, abstract-only, and articles without available full text after personal correspondence were excluded from data retrieval. Additional studies were excluded for the following reasons: the study did not discuss use of antimicrobials prior to the onset of PIGN, did not discuss initial infection, did not discuss PIGN, or data were not reliably extracted or reported.

The following variables were extracted from retrieved studies and recorded in Excel (Microsoft Corp., Redmond, WA, USA): title, author, source, date of study, eligibility or reason for exclusion, study design, sampling procedure, length of follow-up, bias control, statistical methods, setting, region, age and sex of participants, prior infection, intervention with route of delivery, dosage, and length of treatment, development of glomerulonephritis and other sequelae, and participants lost to follow-up.

### 2.3. Statistical Analysis

Contingency tables were created for each included study to evaluate the prevalence of PIGN following an initial bacterial infection. Microsoft Excel and RStudio were then used to calculate the outcome (effect size), standard error, standardized mean difference, and confidence intervals [21]. Heterogeneity was assessed through calculating the inverse variance index (I^2^). The strictly standardized mean difference (SSMD), i.e., the mean divided by the standard deviation of a difference between the two values from each one of the two independent groups, was used to measure the effect size for the comparison between the two groups.

## 3. Results

### 3.1. Antimicrobial Use and Postinfectious Glomerulonephritis

**Figure 1:**
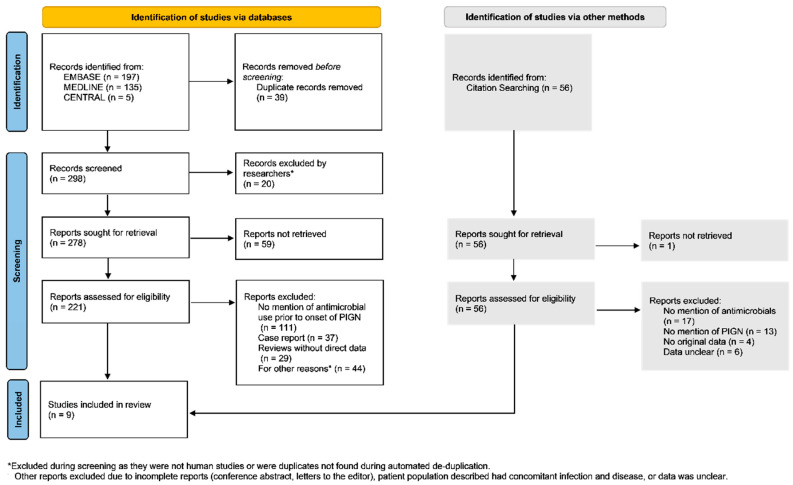
Using PRISMA guidelines for meta-analysis, a flowchart was created to illustrate the sources, inclusion, and exclusion of identified studies from databases and citation searching.

The search for studies concerning antimicrobials and PIGN in humans yielded 298 reports, which were narrowed down to 5 reports (Figure 1). An additional four reports from citation searching were assessed for inclusion eligibility, resulting in nine reports included in the review (Table 1) [22,23,24,25,26,27,28,29,30]. No studies were excluded due to quality concerns. Included studies discussed initial infection occurring at least 7 days prior to any postinfectious sequelae, mentioned the use of or withheld the use of antimicrobial therapy, and mentioned postinfectious glomerulonephritis. Three of nine studies reported incidence of PIGN without prior use of antimicrobial therapies for initial infection. These studies excluded participants who had repeat recent infections, a history of glomerulonephritis, or recent use of antimicrobial therapies. These studies also varied with regard to how initial infections were diagnosed or confirmed. The sample size of these studies ranged from 116 to 4482 patients. Five out of nine studies were conducted outside of the U.S. All nine studies evaluated use of penicillin; four studies utilized oral penicillin, four studies utilized intramuscular penicillin; and one study utilized parenteral penicillin. Two of nine studies were evaluating the efficacy of Penicillin V for group A Streptococcus (GAS) compared to another antimicrobial regimen. Each included study discussed the prevalence of PIGN following an initial infection.

### 3.2. Glomerulonephritis in Patients with Initial Use of Antimicrobial Therapy

The search for studies concerning antimicrobial therapy use prior to development of PIGN yielded eight reports after applying exclusion and inclusion criteria. Studies without cases of PIGN were not considered given the overall rare incidence of PIGN. Significant heterogeneity (I^2^ = 92%, *p* < 0.01) was found in the confidence intervals (CI) of patients initially treated with antimicrobial therapies. This heterogeneity was addressed with random effects analysis. Random effects analysis was utilized based on the assumption that the studies included in this analysis represent a random sample of effect sizes, and the true effect size varies from one study to the next [31].

**Figure 2:**
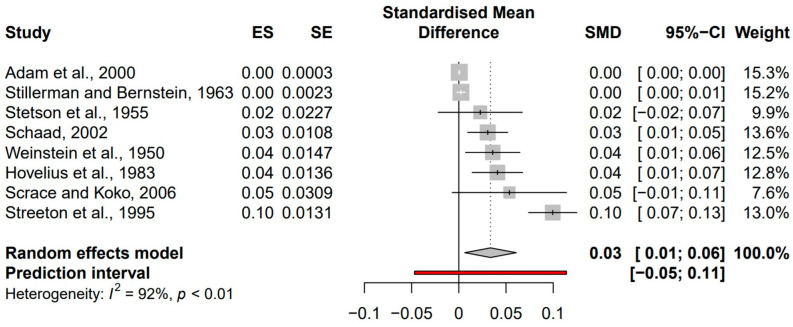
Lists the author and study year, effect size (ES), standard error (SE), standardized mean difference (SMD), and 95% confidence interval (CI) for each study. The forest plot reveals SMD for each study plotted against a random effects model.

The overall standardized mean difference (SMD) in the CI of antimicrobial therapy prior to development of PIGN was 0.03 (95% CI, 0.01 to 0.06). Analysis of these eight studies demonstrated varying effect sizes for PIGN occurring following antimicrobial therapy for an initial infection (Figure 2).

### 3.3. Glomerulonephritis in Patients without Initial Use of Antimicrobial Therapy

The search for studies concerning antimicrobial therapy use prior to development of PIGN yielded three reports where patients were not provided antimicrobial therapies, after applying exclusion and inclusion criteria. Studies without cases of PIGN were not considered given the overall rare incidence of PIGN. Significant heterogeneity (I^2^ = 84%, *p* < 0.01) was found in the confidence intervals (CI) of patients not initially treated with antimicrobials. This heterogeneity was addressed with random effects analysis.

**Figure 3:**
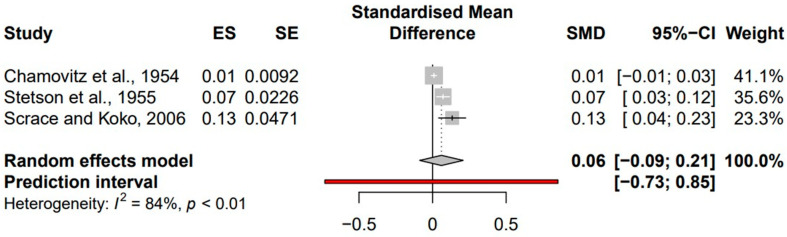
Lists the author and study year, effect size (ES), standard error (SE), standardized mean difference (SMD), and 95% confidence interval (CI) for each study. The forest plot reveals SMD for each study plotted against a random effects model.

The overall SMD of the CI for patients who had not undergone antimicrobial therapy prior to development of PIGN was 0.06 (95% CI, −0.09 to 0.21). Analysis of these three studies demonstrated varying effect sizes for PIGN development following initial infection without antimicrobial treatments (Figure 3).

The SMD for studies where patients were treated with antimicrobials was 0.03 at 95% CI (0.01–0.06) whereas the SMD for studies where patients were not treated with antimicrobials was 0.06 at 95% CI (−0.09 to 0.21).

To measure the effect sizes for the comparison of the two groups, we calculated the SSMD as 0.55.

## 4. Discussion

Overall, our findings reveal a potentially protective effect of antimicrobial therapies on the subsequent development of PIGN. Both types of studies, with and without previous use of antimicrobial therapy prior to development of PIGN, revealed a small SMD. This is likely due to the low incidence of PIGN overall, at around 470,000 cases globally per year [32]. Although PIGN is a rare postinfectious sequela, these results may still provide clinical relevance.

It is worth noting that this study did not evaluate the potential differences between age, sex, or region when calculating the incidence of PIGN as sequelae. The studies included for analysis included participants who developed PIGN at least 7 days following initiation of antimicrobial therapies. Additionally, as seen in Figure 2, Streeton et al. demonstrated a potential outlier with an effect size of 0.10. These authors note that screening can recognize clinically asymptomatic cases, which was also mentioned by Scrace and Koko. This, in combination with mass treatment and availability of penicillin treatments may have prevented the spread of nephritogenic strains of GAS, as they were studying outbreaks in three remote communities.

Potential additive biases also include different time points in treating the initial infection or different antimicrobials and pharmacotherapeutics utilized for treatment. These differences may help explain the considerable heterogeneity observed across the studies. Additionally, differences in addressing patient nonadherence to antimicrobial treatment varied across the studies with subsequent severity of PIGN not addressed.

A previous systematic review published in 2010 evaluated antimicrobials in preventing PSGN [33]. Zaffanello et al. reported that the role of antimicrobials in PIGN prevention is unproven. However, of the nine studies that were included in the review, four appear to be subsets of the same data reported by Adam et al. The study of 4482 patients had the largest dataset, and other publications included in this review of antimicrobials for prevention of PIGN appeared to be subset analyses [22,34,35]. Adam et al. and Scholz concluded that the efficacy of a 5-day antibiotic regimen was equivalent to a 10-day Penicillin V treatment regimen and treatment differences did not result in differences in streptococcal sequelae.

The exact mechanism of how streptococcal infections and subsequent immune responses damage the glomeruli is unknown. However, establishing whether antimicrobials used to treat the initial infection can effectively prevent sequelae is important [1,36,37]. Although GAS infections are generally treated aggressively to prevent the potential future development of rheumatic fever, elucidating the potential of developing other complications, such as PIGN, becomes a more pressing matter as we consider the growing rates of antimicrobial-resistant micro-organisms [38]. Studies on antibiotic efficacy and resistance should inform evidence-based treatment protocols to reduce the risk of refractory infections [39,40].

Although our analysis revealed the presence of considerable heterogeneity, data from this study have revealed potential protective effects of antimicrobial therapy in preventing PIGN. These findings may help further support early treatment of bacterial infections to prevent this type of complication. However, given the analysis of only nine studies, larger cohort studies, likely through global retrospective chart reviews, would be needed to confirm this effect.

## Figures and Tables

**Table 1:** Included studies for this meta-analysis, with information regarding study date, patient ages considered, confirmatory testing of initial infection (if any), type of study (Cohort vs. Randomized Controlled Trial), and number of patients treated or not treated with antibiotics and those that developed PIGN.

Study	Year	Study Location	Type of Study	Date of Study	Population Studied	Type of Sample	Prior Use of Antimicrobials		No Prior Use of Antimicrobials		Total No. of Patients	Antimicrobial Agent Utilized	Infectious Etiology Discussed	Major Findings
							Treatment (*n* = )	Cases (*n* = )	No treatment (*n* = )	Cases (*n* = )				
Adam et al.	2000	Germany	RCT	1995–1998	Ages 1–18	Throat Culture	4482	2	0	0	4482	Oral Penicillin V vs oral macrolides, cephalosporin	GAS	Efficacy of 5-day antibiotic regimen was equivalent to 10 days of penicillin V
Chamovitz et al.	1954	Wyoming, USA	RCT	1953	Not stated (Air Force Base)	No samples taken	257	0	109	1	366	Intramuscular DBED Penicillin	Exudative tonsillitis or pharyngitis	Post-streptococcal sequelae, including glomerulonephritis and rheumatic fever, occurred in control patients whereas none occurred in penicillin treated patients
Hovelius et al.	1983	Sweden	Cohort	1976–1977	All Ages	Throat Culture	220	9	0	0	220	Penicillin V (47%), Erythromycin (9%), amoxicillin or doxycycline (4%)	GAS	PSGN was diagnosed in 9 out of 220 patients, supporting the assumption that early penicillin treatment reduces incidence of PSGN
Schaad et al.	2002	Switzerland	RCT	1996–1999	Ages 2–12	Throat Culture	269	8	0	0	269	Oral Penicillin V vs Oral Azithromycin	GAS	Clinical efficacy of 3-day azithromycin and 10-day penicillin treatments were similar, although had lower levels of bacteriologic eradication
Scrace & Koko et al.	2006	Australia	Cohort	2005	Ages 2–12	No samples taken	56	3	60	8	116	Intramuscular Penicillin	Screening for infected scabies	Screening of children with subsequent treatment using IM penicillin may be an effective community management strategy for APSGN outbreaks
Stetson et al.	1955	Maine, USA	RCT	1952	Not stated (Naval Base)	No samples taken	44	1	140	10	184	Intramuscular Penicillin	Pharyngitis	The incidence of acute nephritis was higher among untreated patients, compared to those receiving early penicillin therapy
Stillerman & Bernstein	1963	New York, USA	RCT	1956–1960	Not stated (pediatric offices)	Throat Culture	442	1	0	0	442	Oral Phenoxymethyl Penicillin	GAS	There was a significantly higher cure rate for nontypeable Streptococcus strains when treated with larger-dose penicillin compaired to smaller-dose treatment.
Streeton et al.	1995	Austrialia	Cohort	1995	Ages 2–14	No samples taken	583	58	0	0	583	Intramuscular Penicillin	Screening for skin infection	Epidemic of APSGN was associated with GAS skin infections. Use of penicillin may have reduced community transmission.
Weinstein et al.	1968	Massachusetts, USA	Cohort	1948	All Ages	Throat and Nose Cultures	167	6	0	0	167	Parenteral Penicillin	GAS	The treatment of scarlet fever with penicillin therapy does not prevent "late" streptococcal sequelae, including rheumatic fever or glomerulonephritis.

Derrick & Dillon excluded in MSD analysis and forest plot creation as PIGN cases occurred within 7 days of initiating antimicrobial treatment. Sholz is a repeat of Adam….

## Data Availability

Not applicable.
